# Tin

**Published:** 1894-10

**Authors:** J. R. Thompson


ARTICLE V.

### TIN.

BY DR. J. R. THOMPSON.

This is not intended for a scientific treatise on the metal named; it is nothing but a sort of unconnected talk, unassuming, and if you expect me to commence at the Welch mines and explain how the metal is obtained from the ore, or to mention all the other places in the world where it can be found, or all the uses it is put to, I am afraid you will be disappointed. I should surely fail if I attempted to do so, for the very good reason that I do not know much about that part of the matter myself, What I am going to try to do is to say something about tin as used in our business.

Now, I used to think that tin foil was pure tin, made into tin foil; but if I am not mistaken, I think I have been told that some of the other metals are present in small proportions, notably, lead. However that may be, I know the tin foil we now get from the dental depots is a most excellent material to use for filling teeth, especially where you want to be as sure of saving the teeth as we can well be. This may be thought of by some of you to be quite an old chesnut I am giving you, and some may say, what is the use of meeting every year to hear the same things over again. To such I say, give us something better yourself, every year, and so make the old chesnuts take a back seat.

To fill a cavity properly with tin foil is very near or quite as difficult to do as to fill it with gold; indeed, it is more difficult, for a great many who use tin very little. Some have told me that they never could use it satisfactory to themselves, or make a filling of it that would save a tooth as well as amalgam. Now, I am not surprised at this from some; but I am very much surprised that more do not use tin. No lazy man will use it much. Why? Because there is more work and less pay in its use than almost any other one thing in the business. But the honest, conscientious, energetic man will use it and plenty of it. Some contend that it wears away too fast, but I think that, again, is so because it is not given a fair test. Try it, and note how long a filling on the grinding surface of a molar will last, if it is well filed, condensed and properly polished and burnished. It will not, probably, last as long as gold or some of the best alloys, but it will last a long time, and, in the majority of cases, it will last until the destruction of the tooth from some other cause. Tin foil is, in my estimation, the very best material we have for saving teeth, everything considered. I mean by this that in proportion to the number of teeth filled with tin, that more of them are saved than those filled with any other material.

Tin is the poor man's filling. Any one can have more work done for the same money by using tin than any other material, quality of work being considered. It is essentially the material of the times. We all know how the people of this country are situated in money matters, and that is why I say that the time suit this material.

Suppose a lady patient comes with all the incisors decayed in the approximal surfaces. From want of money she has allowed them to get wore than she should have done. Now she has got a little money, and comes to you and asks you to do the best you can for her. The six fillings of that size would cost with gold more than twice as much as she has. Will you fill two or three of the largest cavities with gold for her money, or would it not be better to fill them with tin for the same amount? Of course it will be more work for you, by half, for the money, but honor bright, would it not be best, everything considered, to make up your mind to do the work and save the teeth?

If those fillings are put in from the palatine surface very little of the material will show, and the work being done properly the teeth will be saved.

But it will make the tooth dark around the cavity, say some. Well suppose it does. Is the tooth in that condition not better than no tooth? And then in the future we have sound, healthy roots to build a nice, expensive piece of crown and bridge work on.

In looking over an old journal a few days ago, the *Cosmos*, of May, 1889, I noticed an article from Dr. W. D. Miller, Professor in the University of Berlin, Germany, on "Tin and Gold as a filling Material." Abstracts and translations by my old time-friend Dr. Jas. Truman, of Philadelphia. In this article the combination of the two metals are very highly recommended. The preparation is very simple—a sheet each of gold and tin are placed one on top of the other, and then cut in strips and rolled together in ropes or any other form to suit the operator. Some prefer the gold rolled on the outside, others the tin. This combination of the two metals is recommended very strongly indeed and one great merit of the fillings is, that they become very hard with age—becoming so hard in time as to withstand all wear from mastication Another advantage is claimed for it, that after being in the mouth for a short time there is a certain amount of expansion, not enough to make it objectionable, or enough to make an imperfect filling keep out the leakage, but enouge to stop any slight imperfections that may be left in a filling. Now I think that in the combination of the two metals in this way, we will only get the superior qualities of the tin as it goes. I do not mean to say that I do not believe that the two metals should be used altogether, for I do use them together by making the body of tin alone, and then use gold on the grinding surface. But what is to be gained by the use of the two rolled together for the body of the filling, over the tin alone, I must say I am unable to see. The tin is dense enough for the fillings away from the grinding surface, so what is the use of any gold in the body of the filling? It is true that he claims that the two rolled together does not discolor the tooth, but I must say that I am not convinced that this claim can be depended on with impunity. I should think that it would depend entirely which of the two metals came in contact with the walls of the cavity, and if he rolls his material as he says he prefers, (with the tin outside) I must say that I should expect to see the color the same as in a tin filling; but if the gold is rolled on the outside, why then you fail to get the qualities of the tin, and might almost as well put in an all gold filling, except the slight difference in cost would be in favor of the tin.

There is this difference in gold and tin as we usually get them from the depots, (except a few of the so-called soft gold foils.) The difference is this: If you will cut two pieces of tin foil about an inch square, and place them between your finger and thumb,one piece on top of the other piece, you can move them back and forth, thus causing a sliding of one piece over the other. Two pieces of ordinary gold foil will not do this, but will stick together, thus demonstrating the fact that while tin has this quality, ordinary gold foil has not. Now this is just the reason why we can make a better filling with tin than with gold.

The piece of tin next the wall of the cavity is rubbed and burnished close to the wall of the cavity by the next piece sliding past and over it, just as you would burnish a thin piece of platinum over the face of a root if you wanted to make a platinum cap to fit that face perfectly. Of course the pressure on your plugger, with the next piece of tin under it, does the same thing, and you continue until the cavity is full.

What would be the case, under similar circumstances, if you were using gold? Why, instead of one piece sliding past the other, it sticks, and the pressure of your instrument pulls it away from the walls, and the piece following clogs, and will leave a defect at that point. That is why we fail to make a tight filling sometimes, and is the reason why we try to have direct pressure against every wall of the cavity when using cohesive gold. All the force you can put on the gold at that point will not remedy the matter after the clog is there. It is like putting weight on the top of an arch—and when you get enough weight to crush the arch, your tooth will most likely be also crushed. This principle is the old one applied to filling teeth before the cohesive qualities of gold were known. Sometimes dentists would complain of their gold being sticky, and therefore not working well. They then thought this was because the gold was not pure; but the reverse was the case if they had only known it.

So much for gold and tin combined. It may do wonders for some; but for me I prefer them one at a time. I may lack skill—very likely do—but I prefer to give my patient the best in my shop, and I must confess I do not think I could do that with the combination.

I shall now give a few ideas of what others in the profession think on the subject of tin in dentistry.

Tuesday, 10th April, 1888, at the New York Odontological Society, Dr. Bogue says, in an article on "Filling Materials and Methods," where he speaks of tin he calls it "Our old reliable standby," and uses this expression: "But one would use tin for the moderately decayed sixth year molars, and the partially erupted twelfth year molars of his own children, and those of his own children, and those of his best patients who thoroughly trust him, not being sure but those teeth are the ones to last for life, therefore not to be discolored, unduly imperiled or trifled with in any way even by gold operators, at that tender age, nor by amalgam operations, under frail overhanging walls on the grinding surface, when the amalgam being harder than the tooth would cause breakage of those overhanging edges."

*Dental Cosmos*, of January, 1891, page 64, Union Dental meeting in Boston. Paper read by Dr. G. F. Cheney, of St Johnsbury, Vermont. After the reading of the paper, and during the discussion, Dr. G. F. Waters said in relating an experience of over forty years ago—"Filling some cavities in molars in which the decay extended to such a depth that the points of the pulps were exposed. He had placed over the bottom of the cavities, mats made of several thicknesses of tin foil, and had filled the cavities with tin. Some time afterward, at the desire of the patient, he removed the fillings for the purpose of replacing them with gold, and found that there had been an exudation of lime salts, which had formed a natural covering for the pulps, and which he described as being as clear as honey and almost as hard as enamel. The texture of the filling was clearly shown on the surface of the new deposit, proving that the deposit had been made in a fluid state and that the pulp had thus protected itself." Did you ever hear of any other material with such an experience?

I see in *Items of Interest* where a dealer asked a manufacturer of tin foil why the last lot sent him was so much liked and worked so well? The manufacturer told him that it was because there was less tin and more lead in it, and then told how it was made, which was as follows: A piece of tin was placed on each side of a piece of lead, and thus pressed through the rollers until it was thin enough for foil and instead of using pure tin as he and others thought, they were using tin plated lead. The dealer was also told that this material was shipped to a large dealer by the hundred pounds, and that he said that after that he was very careful not to praise the tin foil as pure tin, but that his customers were loud in its praise, and no doubt they thought all the time they were using pure tin.—*Southern Dental Journal.*

